# Performance Evaluation of a Novel Ultrafast Molecular Diagnostic Device Integrated With Microfluidic Chips and Dual Temperature Modules

**DOI:** 10.3389/fbioe.2022.895236

**Published:** 2022-05-19

**Authors:** Shan Lin, Xiaojun Song, Kun Zhu, Quanyu Shao, Yinhang Chen, Wei Cheng, Zhijing Lei, Yu Chen, Yun Luo, Dazhi Jin

**Affiliations:** ^1^ School of Laboratory Medicine, Hangzhou Medical College, Hangzhou, China; ^2^ Key Laboratory of Biomarkers and In Vitro Diagnosis Translation of Zhejiang Province, Hangzhou, China; ^3^ Department of Clinical Laboratory, Laboratory Medicine Center, Zhejiang Provincial People’s Hospital, Hangzhou Medical College, Hangzhou, China; ^4^ Hangzhou Biochip for Diagnosis Technology Co., Ltd., Hangzhou, China; ^5^ School of Biotechnology and Biomolecular Sciences, University of New South Wales, Sydney, NSW, Australia

**Keywords:** molecular diagnostic, ultrafast, microfluidic chip, dual temperature modules, performance evaluation

## Abstract

Ultrafast, portable, and inexpensive molecular diagnostic platforms are critical for clinical diagnosis and on-site detection. There are currently no available real-time polymerase chain reaction (PCR) devices able to meet the demands of point-of-care testing, as the heating and cooling processes cannot be avoided. In this study, the dual temperature modules were first designed to process microfluidic chips automatically circulating between them. Thus, a novel ultrafast molecular diagnostic real-time PCR device (approximately 18 and 23 min for DNA and RNA detection, respectively) with two channels (FAM and Cy5) for the detection of 12 targets was developed. The device contained three core functional components, including temperature control, optics, and motion, which were integrated into a portable compact box. The temperature modules accurately control temperature in rapid thermal cycles with less than ±0.1 °C, ±1 °C and ±0.5 °C for the temperature fluctuation, uniformity, and error of indication, respectively. The average coefficient of variation (CV) of the fluorescence intensity (FI) for all 12 wells was 2.3% for FAM and 2.7% for Cy5. There was a good linear relationship between the concentrations of fluorescent dye and the FIs of FAM and Cy5(*R* ^2^ = 0.9990 and 0.9937), and the average CVs of the Ct values calculated by the embedded software were 1.4% for FAM and Cy5, respectively. The 100 double-blind mocked sputum and 249 clinical stool samples were analyzed by the ultrafast real-time PCR device in comparison with the DAAN Gene SARS-CoV-2 kit run on the ABI 7500 instrument and Xpert *C. difficile*/Epi, respectively. Among the 249 stool samples, the ultrafast real-time PCR device detected toxigenic *C. difficile* in 54 samples (54/249, 21.7%) with a specificity and positive predictive values of 99.0 and 96.3%, which were higher than the Xpert *C. difficile*/Epi values of 94.4 and 88.1% (*p* > 0.05). The ultrafast real-time PCR device detected 15 SARS-CoV-2 positive samples, which has a 100% concordance with that obtained by the DAAN Gene SARS-CoV-2 kit. This study demonstrated that the ultrafast real-time PCR device integrated with microfluidic chips and dual temperature modules is an ultrafast, reliable, easy-to-use, and cost-effective molecular diagnostic platform for clinical diagnosis and on-site testing, especially in resource-limited settings.

## Introduction

Molecular diagnostics plays a crucial role in the early diagnosis of infectious diseases, genetic diseases, and tumors by analyzing gene- or protein-based biomarkers (Biswas, 2016). The immunoassays remain some limitations such as cross reactions, false positive IgM, and the window period at the early stage of infection (Benzigar et al., 2021). Owing to their high sensitivity and specificity, gene diagnostics have been widely used for the rapid diagnosis of infectious pathogens including SARS-CoV-2 (Go et al., 2017; Chen H et al., 2019; Li et al., 2020). Polymerase chain reaction (PCR) tests, especially real-time PCR, have become a popular technology in gene diagnostics (Rödiger et al., 2017). However, the slowdown in PCR tests comes from the time it takes to repeat the heating and cooling processes (Lee et al., 2020).

Based on PCR technology, heating media can be categorized into three types: water bath (Chan et al., 2016), air heated (Qiu et al., 2017), and metal heating block (You et al., 2020). Water bath-based PCR has been abandoned because of its low degree of automation, low amplification efficiency, and lack of consistency (Mahanama and Wilson-Davies, 2021). Air-heated-based real-time PCR has subsequently been developed with air as a heat-transfer medium (Qiu et al., 2019). Although this method increases the amplification efficiency to a certain extent because of the low thermal conductivity and specific heat capacity of air (Miao et al., 2020), additional energy consumption and cost were increased. Metal block-based real-time PCR is currently one of the most widely used products, which utilizes the semiconductor chilling plate to repeatedly heat and cool the metal block for the thermal cycle (Loy et al., 2018). However, temperature-changing process is still needed with about 3°C/s (Wong et al., 2015).

Recently, with the aim of performing real-time PCR tests more efficiently, rapid real-time PCR devices have attracted considerable attention (Romsos and Vallone, 2015; White et al., 2015; Federici et al., 2018) and are of great significance for rapid pathogen detection and on-site diagnosis (Qian et al., 2018). Although some rapid PCR devices have been designed for point-of-care testing (POCT), some technical challenges are still faced, such as complex hardware system design, insufficient miniaturization, and complicated preprocessing (Luppa et al., 2016; Kazuya et al., 2020; Rong et al., 2021). The GeneXpert, developed by Cepheid, is a popular POC device that has been endorsed by the WHO (Heidebrecht et al., 2016), and however it still needs 45–60 min for the test to complete due to one single temperature module conducting the thermal cycle through repeated heating and cooling processes (Stime et al., 2018). Furthermore, novel temperature control methods have been developed to shorten thermocycling time (Rogers-Broadway and Karteris, 2015). Some rapid nucleic acid amplification techniques have been proposed as alternatives to the conventional thermocycling process, including loop-mediated isothermal amplification (Martzy et al., 2017), recombinase polymerase amplification (Lobato and O'Sullivan, 2018), multiple cross displacement amplification (Luu et al., 2021), helicase-dependent amplification (Barreda-García et al., 2018), droplet digital PCR (ddPCR) (Wang et al., 2018) and CRISPR (Dai et al., 2020). Among the above assays, the design of primers is complicated, and false positives can easily arise, making it difficult to provide on-site POC testing (Lau and Botella, 2017).

To address these problems, a low-cost ultrafast molecular diagnostic device was developed with a microfluidic chip and dual temperature modules, which enables rapid PCR by circulating the chip between both of two dual temperature modules through a motion module without the heating and cooling process. In this study, we evaluated the performance of the ultrafast real-time device and detected SARS-CoV-2 and *Clostridioides difficile* (*C. difficile*) to verify its clinical performance.

## Materials and Methods

### Test Equipment

The ultrafast real-time PCR system (CQ100) was offered by Biochip for diagnosis Co., Ltd. (Hangzhou, China).

### Measurement of Temperature Fluctuation, Uniformity, and Accuracy

There are two temperature modules in the CQ100. The target temperature of one module was set to 50, 60, and 65°C, and the other was set to 90, 95, and 100°C. After the set temperature was reached and stabilized, the temperature data of the central points in the two modules were collected using a 50D digital thermometer (Fluke Corp., Washington, DC, United States).

The temperature fluctuation was determined by the temperature differences among different measurement times at the same measured point on the microfluidic chip and was calculated using the formula: ΔT_f_ = ± (T_max_ -T_min_)/2, where ΔT_f_ represents the value of the temperature fluctuation and T_max_ and T_min_ represent the mean maximum and minimum temperatures at the central point of each module for five measurements, respectively.

The temperature uniformity was determined by the temperature difference among various measured points on the microfluidic chip at the same time and was calculated using the following formula: ΔT_u_ = T′_max_ – T′_min_, where ΔT_u_ represents the temperature uniformity and T′_max_ and T′_min_ represent the maximum and minimum temperatures for six measurements at the five points, including one central point and four diagonal points. The concept of the error of indication was introduced to analyze temperature accuracy, which is the temperature difference between the measured and targeted data and can be calculated by the formula: ΔT = T_d_ – T_0_, where ΔT represents the error of indication and T_d_ and T_0_ represent each of the mean temperatures for six measurements at the central point in two modules and the presetting temperature.

### Repeatability, Precision, and Linear Response of Fluorescence Intensity

Two fluorescent channels were set to detect FAM and Cy5 fluorescence in the CQ100, and FAM and Cy5 fluorescence was measured at excitation/emission wavelength settings of 495/518 and 650/670 nm, respectively. The fluorescent dye powder (BiOligo Biotech Co., Ltd., Shanghai, China) was dissolved in distilled water to obtain a 100 μM stock solution. The working solution (8 μL) was added to each well on a microfluidic chip, and a simple program (95°C for 1 s, 65°C for 1 s, 72°C for 1 s, 1 cycle) was set to quickly acquire fluorescence images. The fluorescence intensity (FI) data (Absorbance Unit, a.u.) were obtained based on the gray value of the fluorescent images.

Each dye stock solution was diluted to three concentrations (low, medium, and high) with distilled water to analyze the FI detection repeatability and precision. The FAM solution was diluted to concentrations of 2, 6, and 10 μM, and the Cy5 solution was diluted to concentrations of 0.5, 2, and 6 μM. The inter-well coefficient of variation (CV) was used to evaluate the repeatability, which was obtained from 10 measurements of the FI in the same reaction well. The intra-well CV was used to evaluate the precision, which was determined from the FI among 10 measured reaction wells at the same time. The CV was calculated using the following formula: CV = standard deviation/mean × 100.

Moreover, the dye solutions were two-fold serially diluted to five concentrations ranging from 10 to 2 μM, and then a linear fitting of the relationship between the concentrations and the response FI was performed to evaluate the accuracy of fluorescence acquisition.

### Preparation Detection of Double-Blind Mocked COVID-19 Samples

All included studies were approved by the Ethics Committee of Hangzhou Medical College (ethical approval number: LL2020-03 and LL2020-41). A COVID-19 pseudovirus was constructed as follows. The specific sequences of the N and ORF1ab genes of SARS-CoV-2 (GenBank: MN908947) were synthesized and cloned into the pGEM-T vector (Promega, Madison, WI, United States). After digestion with HindIII restriction enzymes, the target fragments were ligated into the pNCCL1 vector. The capsid protein was expressed in pET-MS2 bacteria after induction with IPTG, and the recombinant proteins self-assembled into virus-like particles that were released into the supernatant and purified by density gradient centrifugation in CsCl. The constructed pseudovirus was validated using Sanger sequencing.

Sputum samples from healthy individuals who underwent a health examination were collected at the Zhejiang Provincial People’s Hospital between 1 March 2020, and 30 June 2020. The mocked sputum samples were prepared by mixing 10 µL of pseudovirus and 190 µL of sputum samples, while sputum samples from healthy individuals were used as negative controls. The prepared double-blind sputum samples were stored at −80°C. RNA from each double-blind sample was extracted using the RNeasy Mini Kit (QIAGEN Inc., Valencia, CA, United States) according to the manufacturer’s instructions. Each sample was divided into two equal parts: one was detected using the CQ100 system, and the other was detected by the DA0990 assay (Daan Gene Co. Ltd., Guangzhou, Guangdong, China) that runs on the ABI7500 instrument (Applied Biosystems Inc., Foster, CA, United States).

### CQ100 Assay

The amplification was performed in a total volume of 10 μL containing 7.5 μL CQ 100 PCR Premix (Biochip for diagnosis) and 2.5 μL sample RNA. The mixture was loaded into each channel of the microfluidic chip, which was snapped into a chip holder and then inserted into the CQ100 device. The reaction was performed under conditions recommended according to the manufacturer’s protocol: reverse transcription at 50°C for 5 min, pre-denaturation at 95°C for 8 s, followed by 40 cycles at 95°C for 7 s and 60°C for 14 s; it takes approximately 23 min, and results were monitored on the screen for the appearance of sigmoidal (S) curves suggesting amplified virus RNA. Positive and negative controls were included in each test run.

### DA0990 Assay

The assay was performed using the DA0990-Detection Kit for 2019-nCoV (PCR-Fluorescence) (DA0990 kit) according to the manufacturer’s instructions. Briefly, the PCR reaction was conducted in 25 μL total reaction volumes containing 17 μL reaction solution A, 3 μL reaction solution B, and 5 μL sample RNA. The mixture was loaded into the PCR tube and was run on an ABI7500 instrument with the following program: reverse transcription at 50°C for 2 min, pre-denaturation at 95°C for 2 min, followed by 42 cycles of denaturation at 95°C for 5 s and annealing and extension at 60°C for 35 s. It took approximately 62 min and was monitored on the screen for the appearance of the S curves. Positive and negative results were included for each test run.

### Collection of Clinical Samples for *C. difficile* Detection

Clinical stool samples were collected from patients with diarrhea at Zhejiang Provincial People’s Hospital between August 1 and 30 December 2020. Liquid, soft, or semi-solid stool samples of sufficient volume were stored at −80°C and transported to Hangzhou Medical College within 48 h for further testing. Each stool sample was divided into two aliquots (1 mL per each), one was analyzed using the CQ100 system, and the other was analyzed by Xpert *C. difficile*/Epi (Cepheid, Sunnyvale, CA, United States). Toxigenic culture (TC) was used as the reference method for evaluation of these two assays as previously described (Neuendorf et al., 2016).

Stool samples were thawed to room temperature (20°C) and genomic DNA was extracted using the QIAamp DNA Mini Kit (QIAGEN), according to the manufacturer’s instructions. The toxin A (*tcdA*) and toxin B gene (*tcdB*) of *C. difficile* were selected as target genes, and sequences were obtained from GenBank. The primers and probes were designed using DNASTAR V5 (DNASTAR, Madison, WI, United States), and the specificity of the primers and probes was verified using the NCBI Primer BLAST database. The primer and probe sequences are listed in Supplementary Table S1. All sequences were synthesized by General Biosystems (Anhui) Co., Ltd.

### CQ100 Assay

Amplification was performed in a total volume of 10 μL containing 5 μL HR qPCR Master Mix (HuiCHem Co., Ltd., Shanghai, China), 2 μL primers and 3 μL sample DNA. The mixture was loaded into each channel of the microfluidic chip, which was then placed into a chip holder and inserted into the CQ100 device. The PCR was performed under the following conditions: pre-denaturation at 97°C for 8 s, followed by 40 cycles at 97°C for 7 s and 61°C for 14 s; it took approximately 18 min, and results were monitored on the screen for the appearance of S curves, suggesting amplified targeted DNA. Each PCR run included both the positive and negative controls.

### Xpert *C. difficile*/Epi Assay

The assay was performed according to the manufacturer’s protocol, as previously described (Xu et al., 2018). Briefly, stool samples were collected with sterile swabs and transferred into reagent-containing sample vials, which were then vortexed for 10 s, and all solutions were introduced into the Xpert *C. difficile* cartridge, and finally placed into the Xpert instrument. The test was performed according to the GeneXpert *C. difficile* assay program.

### Statistical Analysis

The clinical sensitivity, specificity, positive predictive value (PPV) and negative predictive value (NPV) of the CQ100 assay were calculated according to previous studies (Huang et al., 2017). Turn-around time was determined using a single sample, and costs per test were calculated based on the prices of the purchased kits. The 95% confidence interval (CI) was calculated using the SPSS version 19.0 software (SPSS Inc., Chicago, IL, United States); *p* ≤ 0.05, determined by Fisher’s exact test, was considered statistically significant.

## Results

**FIGURE 1:**
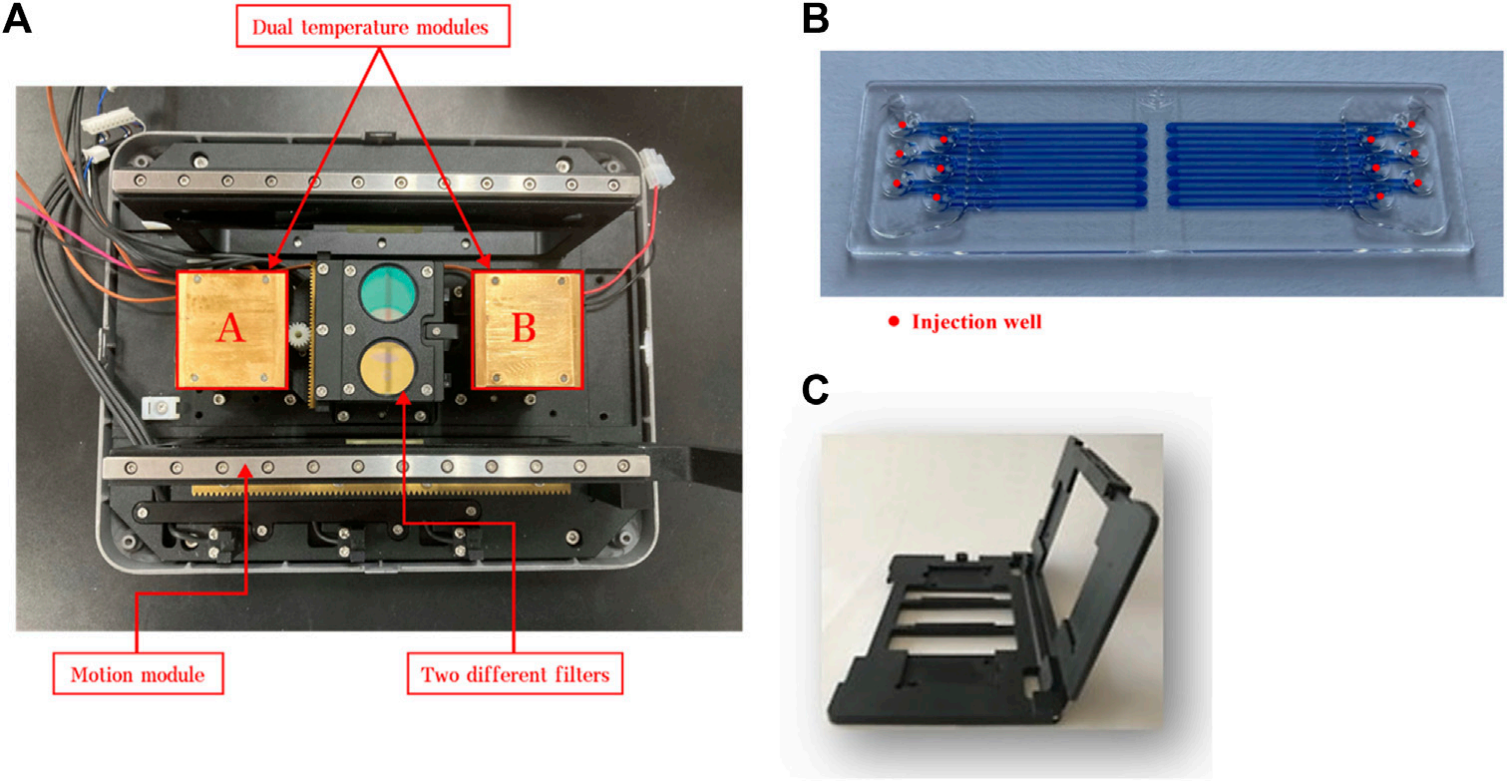
Image of the basic components of CQ100 device: **(A)** dual temperature modules and **(B)** microfluidic chip. The chip circulates back and forth between the module “A” and module “B” to perform the PCR. The target temperature of the module “A” was set to 50°C, 60°C, and 65°C, and the module “B” was set to 90°C, 95°C, and 100°C. **(C)** the matching chip holder.

The CQ100 is a portable real-time PCR device powered by an alternating current supply or a portable battery. The physical dimensions are 269 mm × 209 mm × 206 mm (length × width × height) and the weight are 5.8 kg. This device contains three core functional components that control temperature, optics, and motion. For PCR reactions, a microfluidic chip with 12 channels and a matching chip holder are required, and they are performed under closed conditions to prevent aerosol formation. As shown in Figure 1, the chip circulates back and forth between the module “A” and module “B” to perform the PCR reaction, and fluorescence is monitored when the chip moves to the middle of these two modules. The amplification was shown on the monitor screen, and the Ct values were calculated automatically.

### Evaluation of Temperature Control Performance

**FIGURE 2:**
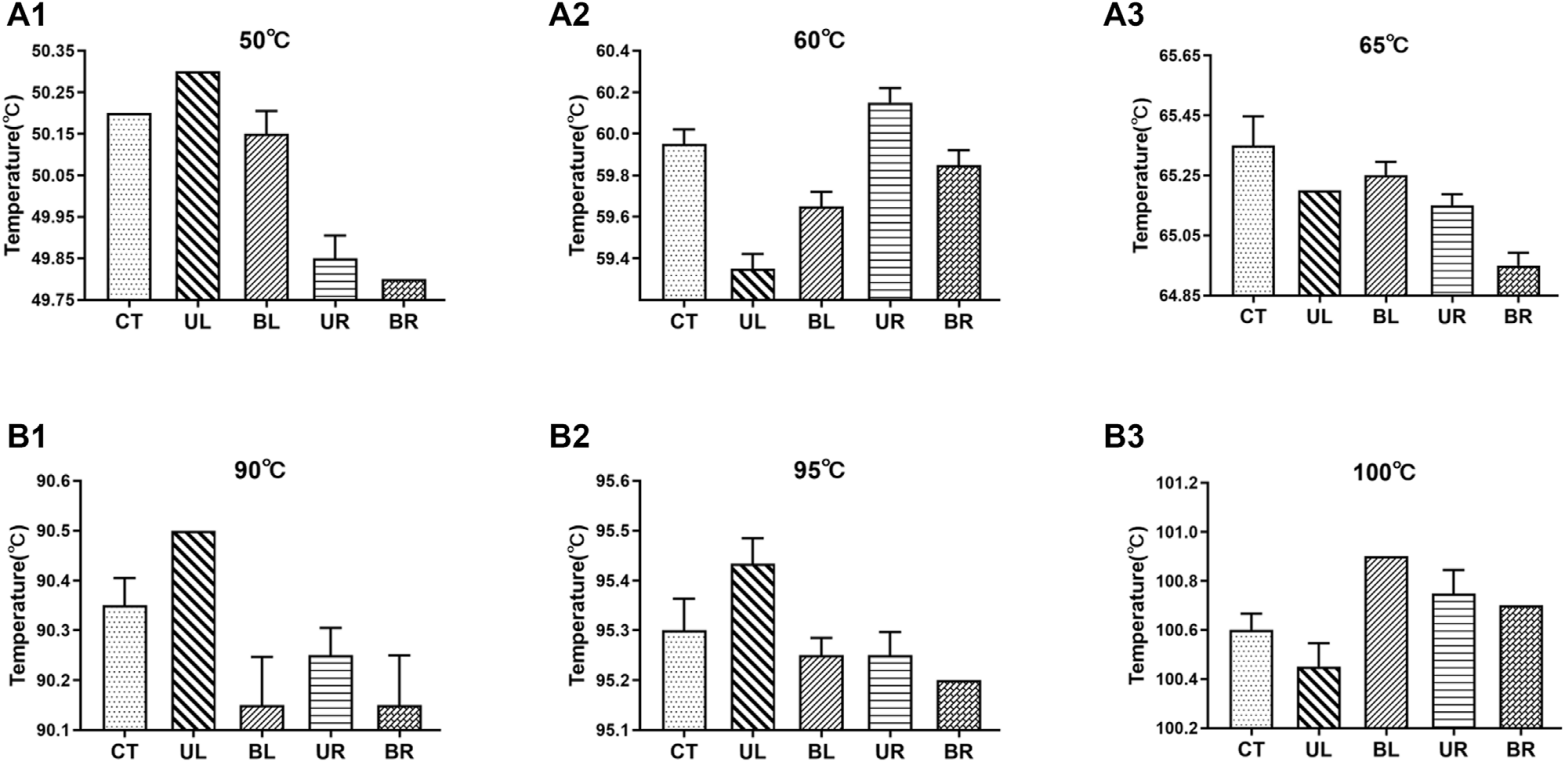
Temperature values at the five measurement points of dual temperature modules. CT, center; UL, upper left; BL, bottom left; UR, upper right; BR, bottom right. **(A1–A3)** the target temperature was set to 50°C, 60°C, and 65°C, respectively; **(B1–B3)** the target temperature was set to 90°C, 95°C, and 100°C, respectively. Values represent the mean ± SD of six measurements of the temperature at each point.

**FIGURE 3:**
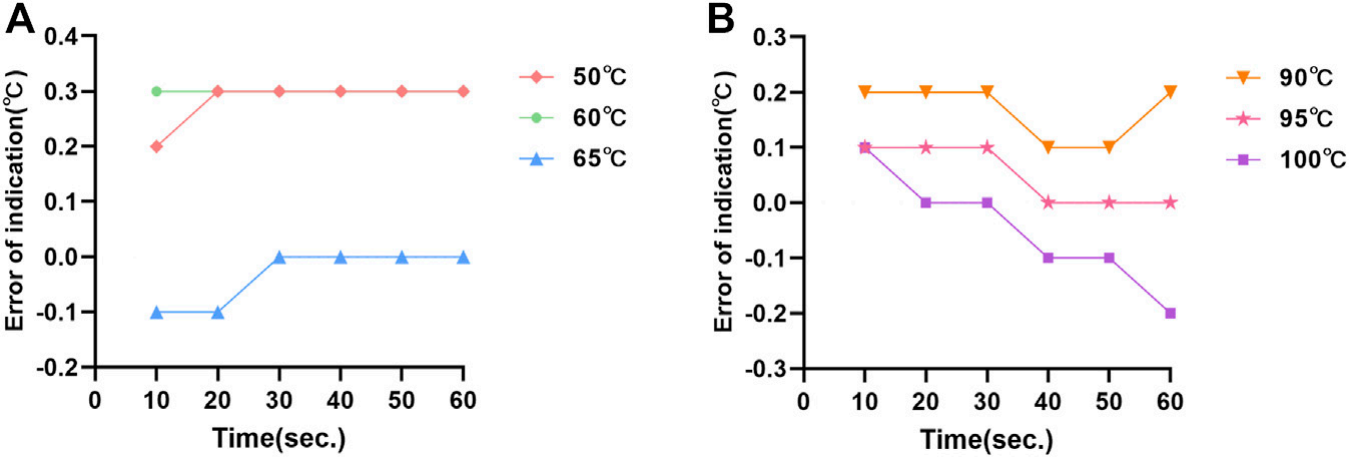
The errors of indication of temperature measurement at the central point of dual temperature modules: **(A)** the errors of indication of the “A” module; **(B)** the errors of indication of the “B” module.

The temperature fluctuations of the dual temperature modules at different target temperatures (50°C, 60°C, 65°C, 90°C, 95°C, and 100°C) were ±0.02°C, ±0.07°C, ±0.01°C, ±0.04°C, ±0.07°C, and ±0.01°C, respectively (Supplementary Table S2). The temperature uniformities of dual temperature modules at each target temperature were 0.5°C, 0.9°C, 0.6°C, 0.5°C, 0.3°C, and 0.6°C, respectively (Figure 2). In addition, the errors of indication of dual temperature modules at each target temperature were 0.28°C, 0.30°C, −0.03°C, 0.17°C, 0.05°C, and −0.05°C, respectively (Figure 3).

### Evaluation of Fluorescence Acquisition Performance

**FIGURE 4:**
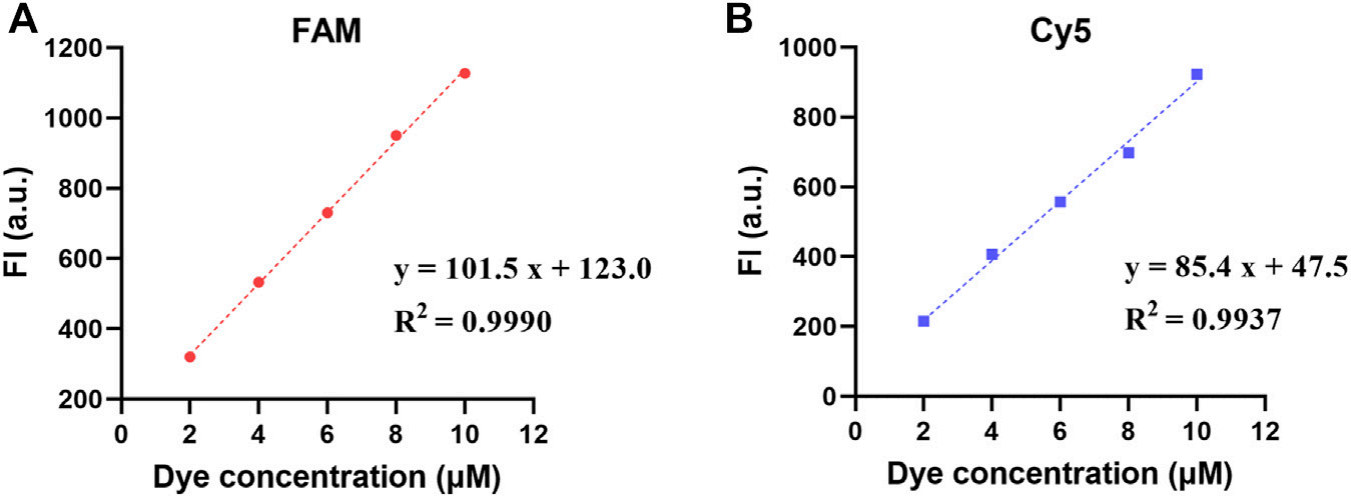
Fluorescence linear analysis in two fluorescence channels: **(A)** the FI of FAM channel; **(B)** the FI of Cy5 channel.

The intra-well repeatability and inter-well precision of the FI at high, medium, and low dye concentrations are shown in Table 1. The intra-well CVs were 1.14–1.65% for FAM and 0.51–2.03% for Cy5. The inter-well CVs were 0.74–3.33% for FAM and 1.86–3.39% for Cy5. Moreover, the FI of the FAM and Cy5 channels had a good linear relationship with the dye concentrations in the range of 2–10 μM, with coefficients of determination (*R* ^2^) of 0.9990 and 0.9937, respectively **(** Figure 4).

**TABLE 1:** Intra-well repeatability and inter-well precision of the FI.

Dye concentration (μM)	FI of intra-well		FI of inter-well	
	X ± SD (a. u.)	CV (%)	X ± SD (a. u.)	CV (%)
FAM	—	—	—	—
2	364.00 ± 4.59	1.26	358.50 ± 10.29	2.87
6	672.50 ± 11.12	1.65	670.50 ± 4.97	0.74
10	1031.50 ± 11.80	1.14	1040.50 ± 34.68	3.33
Cy5	—	—	—	—
0.5	127.00 ± 2.58	2.03	1.50 ± 4.12	3.39
2	412.00 ± 7.15	1.74	416.50 ± 12.03	2.89
6	1143.50 ± 5.80	0.51	1084.50 ± 20.20	1.86

### Detection of Double-Blind Mocked and Clinical Samples

**FIGURE 5:**
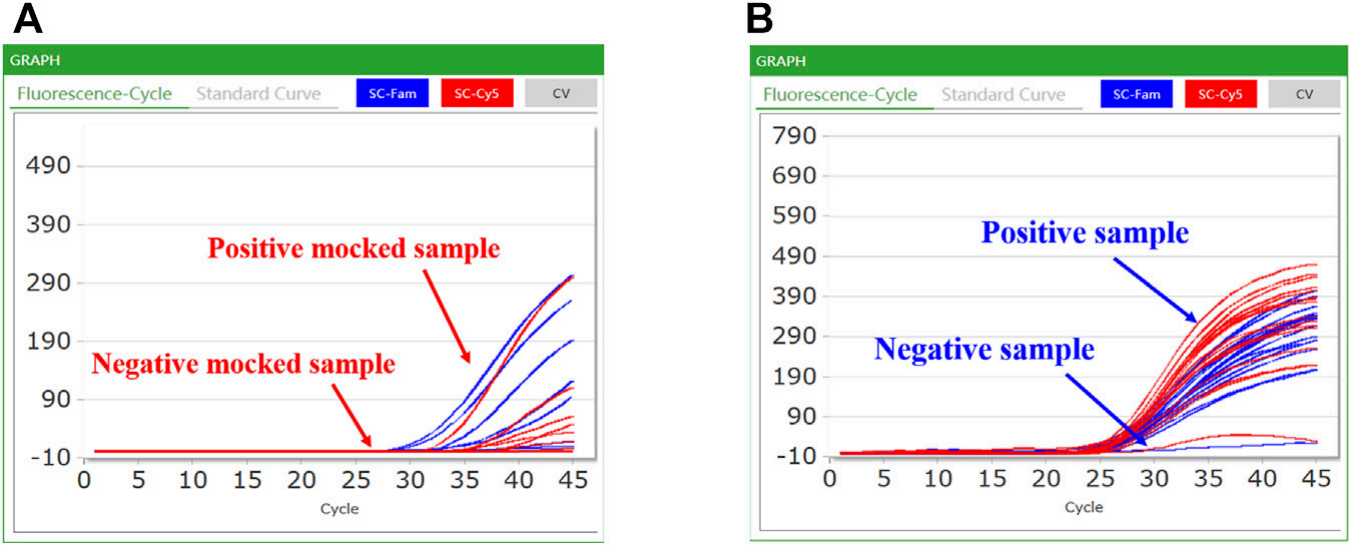
Representative results of the CQ100 assay performed in samples: **(A)** the mocked COVID-19 samples; **(B)** the clinical *C. difficile* samples. A positive sample had an S-shaped curve based on the FAM and Cy5 channel detection and the Ct value of ≤38; in contrast, a negative sample had a differently shaped curve in the FAM and Cy5 channels.

CQ 100 results were reported as positive or negative based on real-time PCR. When a sample did not demonstrate an S-type curve or had a Ct value >38, the result was considered negative; conversely, samples with S-shaped curves and Ct values ≤ 38 were considered positive (Figure 5). Among a total of 100 double-blind mocked sputum samples, the CQ100 device detected SARS-CoV-2 in 15 (15.0%) samples, which shows a 100% concordance with the results obtained by the DA0990 assay. Among a total of 249 stool samples, the CQ100 device detected *C. difficile* toxins in 54 (21.7%), with a sensitivity and PPV of 99.0 and 96.3%, which were higher than the Xpert *C. difficile*/Epi values of 94.4 and 88.1% (*p* > 0.05) (Table 2).

**TABLE 2:** Sensitivity, specificity, and predictive values of the two assays for toxigenic *C. difficile* detection.

Test	No. of samples with indicated results				Sensitivity (%)	Specificity (%)	PPV (%)	NPV (%)
	S^+^T^+^	S^+^T^−^	S^−^T^+^	S^−^T^−^				
CQ100	52	0	2	195	100.0	99.0	96.3	100.0
Xpert *C. difficile*/Epi	52	0	7	190	100.0	94.4	88.1	100.0

S, standard; T, test; +, positive; −, negative; PPV, positive predictive value; NPV, negative predictive value.

### Comparison of the Detection Performance With Other Assays

As shown in Table 3, the reaction volumes, turn-around time (TAT), and cost per test of CQ100 were compared with two other assays issued by the National Medical Products Administration (NMPA). For SARS-CoV-2 detection, CQ100 reaction volumes (8 μL), TAT (23 min, not including RNA extraction), and cost (approximately 5.0 $) per sample were less than those for DA0990 (20 μL, 60 min, approximately 17.2 $). For toxigenic *C. difficile* detection, CQ100 could be completed in 18 min, whereas Xpert *C. difficile*/Epi required 45 min. The CQ100 cost per test (approximately 6.2 $) was 10 times lower than that of Xpert *C. difficile*/Epi (approximately 59.3 $).

**TABLE 3:** Comparison the reaction volumes, TAT and cost per test with other two assays.

	Reaction volumes (µL)	TAT (min)	Cost per test ($)^a^
COVID-19	—	—	—
CQ100	8	23	5.0
DA0990	20	60	17.2
*C. difficile*	—	—	—
CQ100	8	18	6.2
Xpert *C. difficile*/Epi	—	45	59.3

a Cost per test was not an exact cost, because the reagent prices fluctuated continually.

## Discussion

PCR has revolutionized molecular diagnostics for the detection of pathogens (Rajendran et al., 2019). However, conventional real-time PCR systems only have one temperature controlling block, which requires time and energy to perform repeated thermal cycling and limits their application for on-site testing. With the increasing number of emerging infectious diseases worldwide, a variety of molecular-based diagnostic assays has been recently developed to rapidly detect SARS-CoV-2 and *C. difficile*, by which the time to get results ranged from 20 min to 1 h or more (Crobach et al., 2018; Das Mukhopadhyay et al., 2021). Many techniques were used for rapid gene-based assay including isothermal amplification with CRISPR, microfluidic chip with PCR, and etc. Of them, some were expensive, some need large-scale instruments. Thus, there is still an urgent need to develop rapid, inexpensive, and portable molecular diagnostic tools for gene diagnosis.

This study shows that the dual temperature modules integrated into CQ100 have high precision (±0.1°C), uniform temperature distribution (±1°C), and accurate temperature control (±0.5°C). Meanwhile, the fluorescence acquisition had good repeatability and high precision. Thus, it has been demonstrated that CQ100 has promising performance with the high accuracy and reliability for molecular diagnosis. Moreover, CQ100 has high sensitivity and specificity for detecting SARS-CoV-2 and *C. difficile* compared with the golden TC assay, and reduces the TAT by almost 30 min, thereby saving time and expense compared with other two NMPA-cleared assays. However, the detection operation needs to be further optimized. When CQ100 was used to detect *C. difficile* in clinical stool samples, there were discrepant results occurred in 5 samples. These 5 samples were semi-solid, not liquid, the discrepant results between CQ100 and Xpert might be inhomogeneous distribution of *C. difficile* cells. Furthermore, the amount of total genomic DNAs in the wells on the microfluidic chip was lower than that in Xpert. Thus, the further tests should be conducted to confirm these 5 samples using the multi-points sampling method.

With respect to conventional devices, dual temperature modules were firstly designed in the CQ100 combining with the microfluidic chip technology, which conducts PCR reactions by rapidly circulating the chip between both of two dual temperature modules through a motion module and abandon the heating and cooling process. These properties make CQ100 break through the limitations of the reaction speed, which is suitable for on-site detection. Thus, the merit of the CQ100 is summarized as follows: 1) No repeated heating and cooling process is needed in order to greatly shorten turnaround time to get results. 2) The volume of each channel is merely 8 μL in a microfluidic chip, thus reagents and cost were saved per analysis. 3) The compact design and portable construction make CQ100 have the potential to be applied in POC diagnosis.

Recently, rapid PCR research has focused on shortening the thermal cycle time by developing or improving heating methods (Tung et al., 2016; Song et al., 2017; Trauba and Wittwer, 2017; Chen R et al., 2019). Farrar and Wittwer (2015) reported an ultrafast PCR reaction that was based on rapidly changing samples between two water baths, and combined both annealing and extension steps, which was able to complete the amplification within 15–60 s. Kulkarni et al. (2022) demonstrated a continuous-flow microfluidic device that could realize 32 min DNA amplification at an optimum flow rate of 5 μL/min. Although these systems exhibit excellent performance, PCR products need to be analyzed by gel electrophoresis. Furthermore, most currently marketed POC instruments are based on single-temperature zone, such as the Filmarray by BioFire, m-PIMA^TM^ Analyzer by Abbott, and GeneXpert^®^CT/NG by Cepheid, which still require 40–60 min to complete the reaction. Meanwhile, the prices of these machines range from $3,000 to $25,000 (Fernández-Carballo et al., 2018), which makes their availability difficult in resource-limited settings.

CQ100 has been shown to be cost-effective for rapid on-site detection; however, it still has several limitations. First, there were only 12 channels in the microfluidic chip. Second, fluorescence can only be detected in the FAM and Cy5. Third, the nucleic acid extraction step increases the total TAT. Therefore, additional studies should develop a single-site multi-channel microfluidic chip for high-throughput analysis, increase the number of fluorescence channels to achieve multiple detections, and further combined with an automated nucleic acid extraction system to reduce manual operation time.

In summary, this study demonstrates that CQ100 is an ultrafast, affordable, and portable molecular diagnostic tool for pathogen detection. Additionally, dual temperature control heating–based real-time PCR provides a new paradigm for rapid molecular diagnosis of a variety of infectious diseases.

## Data Availability

The original contributions presented in the study are included in the article/**Supplementary Material**, further inquiries can be directed to the corresponding authors.
