# Supplementary material for: Performance Evaluation of a Novel Ultrafast Molecular Diagnostic Device Integrated With Microfluidic Chips and Dual Temperature Modules
Source: Front Bioeng Biotechnol. 2022 May 19;10:895236. doi: 10.3389/fbioe.2022.895236 (PMC9162139; doi:10.3389/fbioe.2022.895236)
Supplement: Supplementary file 1 [file DataSheet1.docx]

Supplementary Material

# Supplementary Table 1 Primer and probe sequences

| Primer/probe | Sequences (5′→3′) | Target genes |
| --- | --- | --- |
| tcdA-F | TCCTAATGCTCCTTCAAGAGAGTT | *tcdA* |
| tcdA-R | CTAGTTCCGTCATCTTCCAATGA |  |
| tcdA-P | Cy5-CCTGGAACTGCTCCAGTATCCCACC-BHQ2 |  |
| tcdB-F | AATGGTATTACCTAATGCTCCAGATA | *tcdB* |
| tcdB-R | CAGTTTTGTGCCATCATTTTCTAA |  |
| tcdB-P | FAM-CCTGGTGTCCATCCTGTCTCACAAGC-BHQ1 |  |

# Supplementary Table 2 Temperature fluctuations at the central point of dual temperature modules

| Target  temperature (℃) | T_max_ (℃) | T_min_ (℃) | ΔT_f_ (℃) |
| --- | --- | --- | --- |
| 50 | 50.26 | 50.22 | ± 0.02 |
| 60 | 60.26 | 59.80 | ± 0.07 |
| 65 | 65.02 | 65.00 | ± 0.01 |
| 90 | 90.06 | 89.98 | ± 0.04 |
| 95 | 95.26 | 95.12 | ± 0.07 |
| 100 | 100.30 | 100.28 | ± 0.01 |
